# Bioequivalence Studies of Vitamin D Gummies and Tablets in Healthy Adults: Results of a Cross-Over Study

**DOI:** 10.3390/nu11051023

**Published:** 2019-05-07

**Authors:** Carol L. Wagner, Judy R. Shary, Paul J. Nietert, Amy E. Wahlquist, Myla D. Ebeling, Bruce W. Hollis

**Affiliations:** 1 Department of Pediatrics, Shawn Jenkins Children’s Hospital, Medical University of South Carolina, 165 Ashley Avenue, MSC 917, Charleston, SC 29425, USA; sharyj@musc.edu (J.R.S.); ebelingm@musc.edu (M.D.E.); hollisb@musc.edu (B.W.H.); 2 Department of Public Health Sciences, Medical University of SC, 135 Cannon Street, Charleston, SC 29425, USA; nieterpj@musc.edu (P.J.N.); herrin@musc.edu (A.E.W.)

**Keywords:** vitamin D, cholecalciferol, bioequivalence, bioavailability

## Abstract

The objective of this investigation was to compare bioavailability between single oral dose Vitamin D_3_ (vitD_3_) gummies vs. tablets in healthy adults. An initial crossover, randomized clinical trial involving healthy adults (*n* = 9) was conducted followed by a larger, confirmatory study (*n* = 31). Healthy participants aged 18–45 years with body mass index (BMI) 18–30 without anemia or vitD deficiency were randomized to receive 20,000 international units (IU) vitD_3_ as single dose gummies or tablets with serial samples obtained to measure plasma vitD_3_ at baseline, 3, 6, 10, 24, and 48 h followed by a 2-week washout period. The same participants then crossed over to receive 20,000 IU vitD_3_ in the form not previously given, with sampling at the same time points. Deidentified blood samples were analyzed for vitD_3_ concentration by liquid chromatography (LC)-mass spectroscopy. In Study 1, results suggested bioavailability was greater with gummies compared with tablets, (effect size 1.08 at 24 h). In Study 2, the area under the concentration curve (AUC) was higher with gummies than tablets (gummy mean (95% CI): 1474 ng·/mL (1393–1555); tablet mean (95% CI): 774 ng·h/mL (693–855), *p* < 0.0001). Average peak blood concentration (C_max_) values were significantly higher with gummies (gummy: 47.3 ng/mL; tablet: 23.4 ng/mL; *p* < 0.0001). VitD_3_ gummies had greater bioavailability than tablets with higher vitD concentrations over time, which may have implications for achieving vitD sufficiency.

## 1. Introduction

There is increasing evidence that vitamin D supplementation is essential in providing adequate delivery of this preprohormone implicated not only in calcium metabolism but also in immune function [1,2]. Historically, the main source of vitamin D was through the interaction of ultraviolet B from sunlight with 7-dehyrocholesterol in the epidermis of the skin that resulted in the synthesis of vitamin D through a secondary thermal reaction in the skin. The dependence on sunlight exposure for vitamin D in humans has not diminished, but due to limited time spent outdoors or the use of protective clothing or sunscreens affecting sunlight exposure, there has been an increase in vitamin D deficiency states that are linked with significant health consequences [3,4]. Supplementation is seen as a viable option to provide individuals with adequate vitamin D stores, yet compliance or adherence with vitamin D supplementation is often in question [5]. In our previous studies with pregnant and lactating women, adherence to vitamin D supplementation using tablets varied, and for those who completed these studies adherence was 69–75% [6,7]. Difficulties with adherence in taking vitamin D tablets also have been reported in other studies, compromising the results of intention to treat trial design [8,9,10]. In a focus group conducted by our group at the end of two vitamin D supplementation during pregnancy studies, participants shared that the types of pills taken impacted their adherence to taking the supplements. While vitamin D in the form of chewable tablets or gummies may improve adherence or compliance with taking daily vitamin D, the question has been posed if such formulations have similar bioequivalence and bioavailability to that of traditional tablet formulations.

Bioequivalence is the property wherein two drugs with identical active ingredients or two different dosage forms of the same drug possess similar bioavailability and produce the same effect at the site of physiological activity. The US Food and Drug Administration (FDA)’s definition of bioequivalence is pharmaceutical equivalents whose rate and extent of absorption are not statistically different when administered to patients or subjects at the same molar dose under similar experimental conditions [11].

Given these definitions, the objective of this initial pilot and then the larger, confirmatory study was to determine whether two vitamin D_3_ (cholecalciferol) preparations (gummy vs. tablet) were bioequivalent. It was hypothesized that the bioavailability of vitamin D_3_ gummies and tablets would be equivalent as defined by peak blood concentration (C_max_) and the time the peak concentration (T_max_) was achieved.

## 2. Methods

### 2.1. Study Design and Setting

Both Studies 1 and 2 were IRB-approved, single center, crossover, randomized, comparator controlled, and single blind (examiner-blind with blood sample ID) clinical trials. Specifically, the initial clinical trial was approved by the Medical University of South Carolina’s (MUSC’s) Office of Research Integrity (ORI; PRO 00059769) and was registered with clinicaltrials.gov (NCT03552666) with the larger, confirmatory study (Study 2) listed under the same ORI number with a second clinicaltrials.gov registration number (NCT03552653). All subjects were evaluated in the Research Nexus at MUSC, an outpatient research center. The procedures followed were in accordance with the ethical standards of MUSC’s ORI on human experimentation and in accordance with the Helsinki Declaration of 1975 as revised in 1983. Each participant signed a written informed consent prior to his/her participation.

#### 2.1.1. Study Population

Healthy adults between the ages of 18 and 45 years old were eligible to participate as long as the other inclusion and exclusion criteria were met (Table 1).

#### 2.1.2. Time of Participant Recruitment and Conduct of Studies 1 and 2

The initial, pilot study (Study 1) was conducted from January 2, 2017, through February 28, 2017. The larger, confirmatory study (Study 2) was conducted from August 26, 2017, through November 22, 2017.

#### 2.1.3. Washout Period between Vitamin D Formulation Exposures

Two weeks was chosen as the necessary time between dosing as that was more than five half-lives for elimination of the parent compound vitamin D from circulation.

#### 2.1.4. Vitamin D Preparations and Dosing for Studies 1 and 2

Participants were randomized to receive either the gummy or the tablet formulation first (Phase 1) with a two-week washout period at which time the participants crossed over to receive what they had not received the prior time (Phase 2). The gummies were manufactured by VitaFusion (Church & Dwight, Princeton, NJ); the excipients (vehicle/filler substances) were tapioca syrup, sucrose, water, and gelatin; less than 2% of: canola, lecithin, citric acid, color (purple carrot juice concentrate), fractionated coconut oil (contains beeswax and/or carnauba wax), malic acid, medium chain triglycerides, natural flavor, pectin, and sodium citrate. The tablets were manufactured by Nature Made (Mission Hills, CA); the excipients in the tablets were cellulose gel, maltodextrin, stearic acid, gelatin, magnesium stearate, croscarmellose sodium, and corn starch.

#### 2.1.5. Study Protocol for Studies 1 and 2

An IRB approved telephone screener was used for telephone recruitment of potential subjects from an MUSC subject database created when potential subjects contacted the recruitment group for participation in the study. If potential subjects met initial inclusion criteria, then a screening visit was set up to obtain screening laboratory parameters.

#### 2.1.6. Screening Visit

Screening visits were scheduled after obtaining written, informed consent. A whole blood sample was drawn and processed for analysis. All eligible subjects were notified of screening results, and, if taking any exclusionary medications, started a 14-day washout period prior to the first dosing day. There was a two-week washout period if a subject had been on a vitamin D preparation at the time of recruitment. This washout period addressed the exclusion criteria of: (a) Use of vitamin D, multivitamins containing vitamin D, foods or beverages fortified with vitamin D and/or other natural health products containing vitamin D, and (b) consumption of grapefruit/grapefruit juice within 14 days of randomization and during the study.

#### 2.1.7. Phase 1 Study Visit

Each participant had a baseline whole blood sample drawn to be processed and plasma frozen until batch measurement of circulating vitamin D_3_ was performed. At time 0, a pretreatment blood sample was obtained and then each participant was randomly assigned by a computer-generated block-design program to receive during Phase 1 either 20,000 international units (IU) ± 2.5% gummy vitamin D_3_ preparation (9.5 gummies) or tablet vitamin D_3_ preparation (5.75 tablets) as a single dose.

All gummies/tablets were administered by the study coordinator to participants. Instructions were given that the gummies should be chewed, not swallowed whole, and to be taken with an 8-ounce glass of water within 2 min. Tablets were to be swallowed whole, and to be taken with an 8-ounce glass of water within 2 min. Compliance was assured as all subjects were provided the quantity of vitamin and took the assigned dose orally in the clinic in the presence of the study coordinator following use instructions per label. After administration of the vitamin D formulation, each participant was given a standardized breakfast at time 30 min.

Serial whole blood samples were drawn at time 0 (baseline; prior to administration of the vitamin preparation), and then at 3, 6, 10, 24, and 48 h following administration of the vitamin preparation. All blood samples were collected by research nurses in Research Nexus outpatient clinic at baseline (before treatment, considered time 0) and then at the appointed times ± 15 min. Whole blood samples were processed, plasma removed, aliquoted in duplicate, and frozen at −80 °C until batch shipped as blinded samples to Heartland Assays, Ames, Iowa for analysis.

#### 2.1.8. Phase 2 Study Visit

Following the 2-week washout period, subjects reported to the research outpatient facility and had a second baseline (time 0) blood draw. The participant then received 20,000 IU vitamin D_3_ in the form not previously dosed, with whole blood sampling at the same time points as detailed for Phase 1 of the study.

#### 2.1.9. Laboratory Measurements

##### Screening Laboratory Values

As part of the screening entry criteria, measurement of hemoglobin and serum calcium was performed in a CLIA-certified laboratory at MUSC.

##### Vitamin D Content of Gummy and Tablet Formulations

Independent validation of the content of the gummy and tablet formulations of vitamin D_3_ was performed by Heartland Assays (Ames, IA). Five of each preparation type (gummy and tablet) were analyzed and the mean ± SD reported to the investigators as described here:

For Study 1, Heartland Assays reported vitamin D_3_ content of the two preparations: Vitamin D content was measured analytically prior to dosing as: 2091.7 ± 115 IU per gummy and 3527.8 ± 467 IU per tablet. The number given to each subject was 9.5 gummies and 5.75 tablets to meet the target dosage of 19,500–20,500 IU. At the end of Study 1 on 2/21/2017, vitamin D content was measured again analytically as 1750.6 ± 55 IU per gummy and 3519.1 ± 815 IU per tablet to ensure the amount taken.

For Study 2, Heartland Assays reported vitamin D_3_ content of the two preparations: Vitamin D content was measured analytically prior to dosing on 9/15/2017 as: 1729.9 ± 14.9 IU per gummy and 4276.6 ± 467.5 IU per tablet. The number given to each subject was 11.75 gummies and 4.75 tablets to meet the target dosage of 19,500–20,500 IU. At the end of the study on 1/22/2018, vitamin D content was measured again analytically as 1696.6 ± 27.0 IU per gummy and 3784.6 ± 307.6 IU per tablet to ensure the amount taken was in the range required.

##### Screening serum 25(OH)D measurement

Serum 25(OH)D concentration at screening was measured by radioimmunoassay (Diasorin, Stillwater, MN) in the laboratory of Hollis/Wagner. Internal control standards were run with each assay to control for intra-assay variation, which was <10% throughout the study.

##### Serial Measurements of circulating plasma vitamin D_3_

Circulating plasma vitamin D_3_ of each de-identified sample was measured by Heartland Assays (Ames, IA) as a single run in duplicate by LC-mass spectrophotometry upon completion of participant dosing and blood draws to minimize inter-run variability. The total circulating plasma vitamin D_3_ at time 0 (baseline), 3, 6, 10, 24, and 48 h was measured for each subject, from which the mean (SD) was calculated for the two treatment groups: gummies vs. tablets.

#### 2.1.10. Raw Data Handling

The data regarding sociodemographic and clinical data from MUSC were entered directly into an Excel spreadsheet. The raw vitamin D data from Heartland Labs was sent via email and entered into an Excel spreadsheet with double entry verification.

### 2.2. Statistical Methods

The primary outcome measures quantified the extent of systemic absorption as defined by area under the concentration curve (AUC) from time 0 to time 48 h and the maximum concentration achieved during the 48-h observation period (C_max_). Secondary outcome measures focused on the rate of systemic absorption and included the time point when the peak concentration (T_max_) was achieved along with the change in total circulating vitamin D_3_ from baseline at the various time points. These metrics are recommended by the FDA when reporting on bioequivalence and bioavailability studies [11].

The mean and standard deviation of circulating vitamin D_3_ at each time point (0, 3, 6, 10, 24, and 48 h) were calculated separately for gummies vs. tablets. To quantify differences between the gummy and tablet preparations at each time point, Cohen’s *d* effect sizes were determined. To mitigate any baseline differences, these effect sizes were calculated as the mean change from baseline during the gummy phase minus the mean change from baseline during the tablet phase, all divided by the standard deviation of the differences.

In each study, the AUCs were compared between the gummy and tablet phases using linear mixed effects (LME) models. The LME models initially included a main effect for preparation (gummy vs. tablet), sequence effect (gummy then tablet vs. tablet then gummy), phase effect (phase 1 vs. phase 2), and baseline vitamin D_3_. The models also included random subject effects to account for within-subject correlation. For both studies, no significant sequence effects or phase effects were noted in the LME models, and thus, these effects were removed from the final models.

As suggested for bioequivalence studies, geometric mean ratios along with 90% confidence intervals were calculated for each outcome to quantify overall differences between gummy and tablet preparations [11]. For Study 2, we also investigated whether the findings were consistent between men and women, and between whites, Hispanics, and other ethnicities. Data were analyzed using SAS 9.4 software (Cary, NC).

### 2.3. Sample Size and Power Considerations

In Study 1, the sample size (*n* = 9) was selected so that the study would be large enough to provide estimates of bioavailability to inform the next step for the larger, definitive study. In Study 2, the sample size was based on data from the pilot study. Specifically, in the pilot study, the unadjusted arithmetic means for the AUC of 1305.6 ng·h/mL (gummy) and 1040.4 ng·h/mL (tablet) were seen in a sample size of *n* = 9, with a mean difference ± standard deviation of 265.1 ng·h/mL ± 194, providing an effect size of 1.37. A relatively similar effect size was observed for C_max_ (1.15). Assuming the true difference between gummy and tablet was as small as the lower 95% confidence limit for the unadjusted arithmetic mean difference of the AUC, the effect size between groups was considered to be as small as 0.6. Since the findings from Study 1 suggested a possibility that the gummy preparation yielded superior bioavailability when compared to tablet, Study 2 was designed to detect a difference in bioavailability between groups. Based on the data generated in Study 1, in order to detect an effect size as small as 0.6 to show superiority of the gummy, it was recommended that a sample size of *n* = 24 be enrolled. This sample size would provide at least 80% power using a linear mixed effects model framework comparing the two groups with α = 0.05 and 2-sided hypothesis testing. In order to ensure that there would be sufficient sample size (*n* = 24) at the completion of the study projecting a liberal attrition rate of 30% (screen fails, drop out), a sample size of *n* = 31 was chosen.

## 3. Results

### 3.1. Study 1

**Figure 1:**
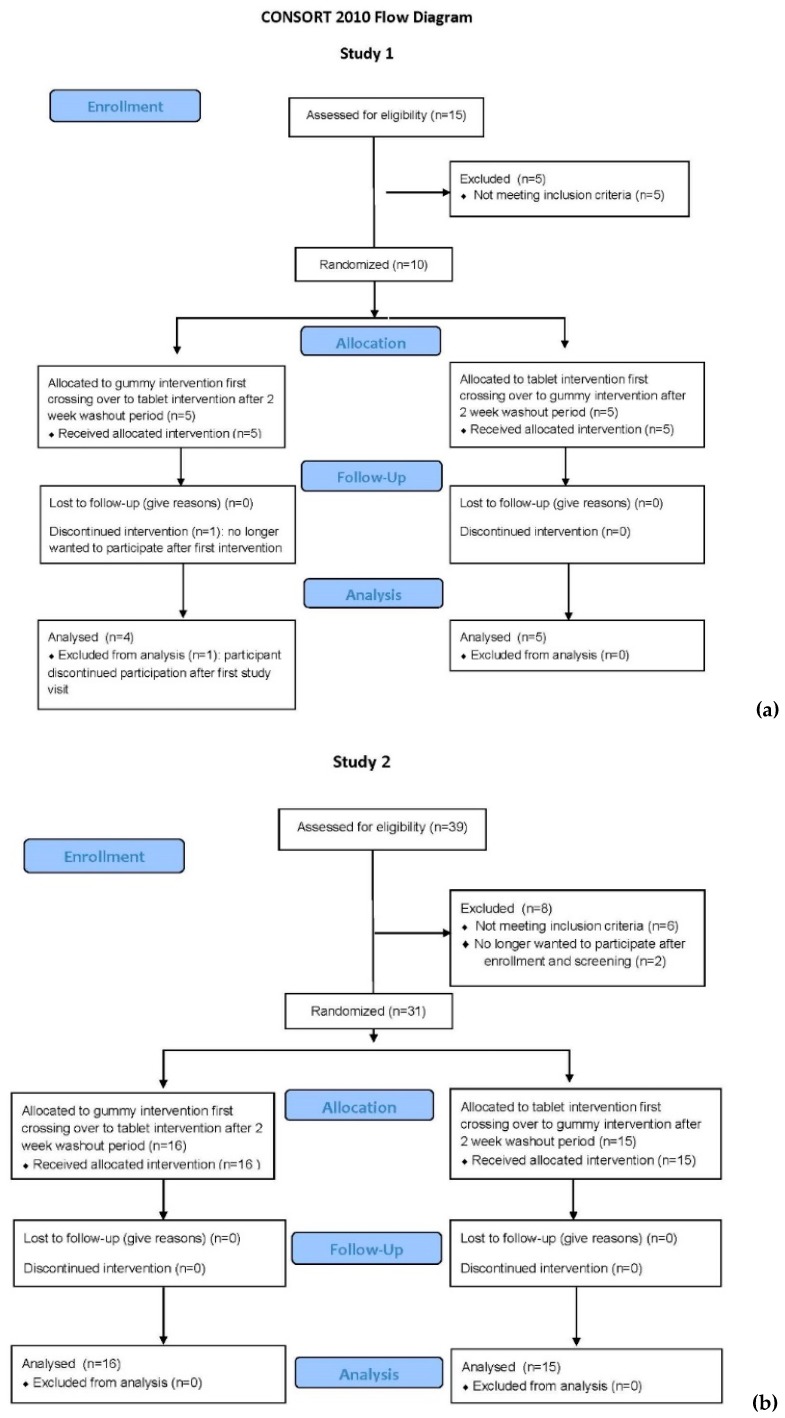
CONSORT flow diagrams of Studies 1 (**a**) and 2 (**b**).

As shown in the CONSORT (Consolidated Standards of Reporting Trials) Flow Diagram for Study 1 (Figure 1a), there were 15 participants who consented to participate in this bioequivalence trial: 8 females and 7 males. Of those, there were 5 screen fails: 1 Caucasian female for hemoglobin <12 g/dL, 2 African–American females with body mass index (BMI) >30; and 2 African–American male subjects with a total circulating 25(OH)D <25 ng/mL (62.5 nmol/L). All pregnancy tests of the female participants were negative at the time of enrollment and study visits. One subject (Caucasian male) withdrew after initial consent and first dosing; the reason given was that he was no longer interested in participating. Nine participants completed the study. The sociodemographic characteristics of those nine individuals are summarized in Table 2.

**Figure 2:**
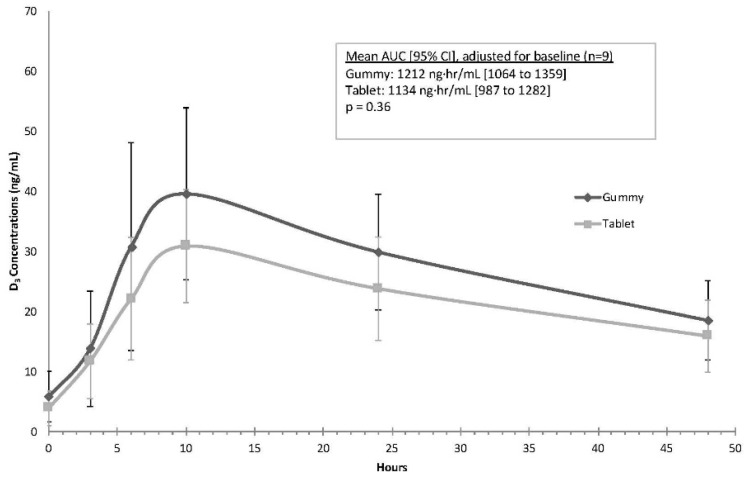
Time course for Study 1 of parent compound vitamin D_3_ over time expressed as the mean ± standard deviation in ng/mL gummy vs. tablet preparation (*n* = 9).

Baseline vitamin D_3_ concentration (mean ± standard deviation; mean ± SD) differed between the groups (gummy: 5.8 ± 4.2 ng/mL, tablet: 3.9 ± 2.9 ng/mL, *p* = 0.03), and therefore, all analyses comparing concentration levels were adjusted for baseline vitamin D_3_ concentrations. Figure 2 illustrates the concentration curves for each of the preparations. After adjusting for baseline differences in vitamin D_3_, the mean AUC was higher with gummies when compared to tablets, but this finding was not statistically significant (gummy mean (95% CI): 1212 ng·h/mL (1064 to 1359); tablet mean (95% CI): 1134 ng·h/mL (987 to 1282), *p* = 0.36). The average C_max_ values were similar during the two phases after adjusting for baseline vitamin D_3_ (gummy: 36.9 ng/mL (32.0 to 41.9); tablet: 33.6 ng/mL (28.6 to 38.6); *p* = 0.35). The time to maximum concentration (T_max_) was 10 h for all participants with both formulations. Table 3 lists the mean ± SD changes in vitamin D_3_ concentrations from baseline, along with effect sizes comparing the two preparations at the various time points; consistent with Figure 2, the largest differences between the gummy and tablet phases occurred at 10 (*d* = 0.87) and 24 h (*d* = 1.09).

After adjusting for baseline concentrations, the geometric mean ratio for the AUC indicated that mean concentrations were higher in the gummy phase (gummy: tablet ratio = 1.20, 90% CI: 1.07 to 1.35). Similarly, the geometric mean for C_max_ was higher in the gummy phase (gummy: tablet ratio = 1.23, 90% CI: 1.06 to 1.43).

### 3.2. Study 2

As shown in the CONSORT Flow Diagram for Study 2 (Figure 1b), there were 39 participants who signed the consent to participate in Study 2: 20 females and 19 males. Of those, there were 6 screen fails: 1 Caucasian male and 1 Hispanic male for hemoglobin concentration < 14.0 g/dL, and 4 subjects (1 Indian male, 1 Hispanic female, 1 Caucasian male, 1 African–American male) with a total circulating 25(OH)D <25 ng/mL (62.5 nmol/L). All pregnancy tests of the female participants were negative at the time of enrollment and study visits. Two subjects (Caucasian females) withdrew after initial consent and screening; the reason given was that they were no longer interested in participating. Thirty-one participants completed the study. The sociodemographic and clinical characteristics of the cohort are summarized in Table 4.

Unlike in Study 1, in Study 2 there was no difference in mean baseline vitamin D concentration between the gummy and tablet groups. All participants received both doses of vitamin D between October 2, 2017, and November 20, 2017, and the 2-week wash-out period was followed between dosings for all participants.

**Figure 3:**
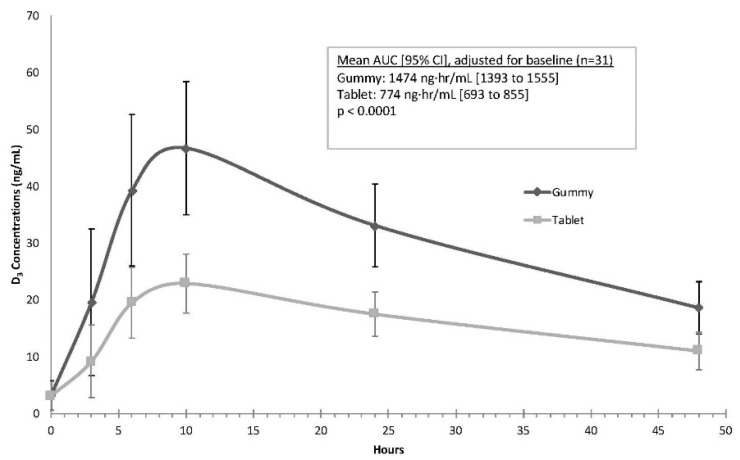
Time course for Study 2 of change in vitamin D_3_ over time expressed as the mean ± standard deviation in ng/mL gummy vs. tablet preparation (*n* = 31).

As shown in Figure 3, the mean gummy serum vitamin D concentrations along a time course of 3, 6, 10, 24, and 48 h were consistently higher than serum concentrations after tablet dosing. Results of the mixed model indicated that the mean AUC was higher for the gummy phase when compared to the tablet phase, a finding that was highly statistically significant (gummy mean (95% CI): 1474 ng·h/mL (1393 to 1555); tablet mean (95% CI): 774 ng·h/mL (693 to 855), *p* < 0.0001). The average C_max_ values also were significantly higher during the gummy phase (gummy: 47.3 ng/mL (44.4 to 50.2); tablet: 23.4 ng/mL (20.5 to 26.4); *p* < 0.0001). The mean (SD) T_max_ for both the gummy and tablets was similar at 9.7 (3.1) for gummy and 9.5 (3.2) for tablet preparations (*p* > 0.9). Table 5 lists the changes in vitamin D_3_ concentrations from baseline, along with effect sizes comparing the two preparations at the various time points; consistent with Figure 3, large differences between the gummy and tablet phases starting at 3 h (*d* = 1.02) and continuing through 48 h (*d* = 2.3) were noted.

The geometric mean for the vitamin D_3_ AUC curve was significantly different between the groups with higher concentrations following gummy ingestion when compared to tablet ingestion (gummy: tablet ratio = 2.12, 90% CI = 1.98 to 2.26, *p* < 0.001). Similarly, the geometric mean for C_max_ was significantly higher following gummy ingestion when compared to tablet ingestion (gummy: tablet ratio = 2.16, 90% CI = 1.98 to 2.35, *p* < 0.001).

When analyzed by race/ethnicity, the directionality of the findings persisted, with significantly (*p* < 0.05) higher vitamin D concentrations (i.e., for both AUC and C_max_) during the gummy vs. the tablet phase for whites/Caucasians (*n* = 22), Hispanics (*n* = 6), and other ethnicities (*n* = 3). Similarly, when analyzed by gender, both males and females had significantly (*p* < 0.05) higher vitamin D concentrations (i.e., for both AUC and C_max_) during the gummy phase compared to the tablet phase.

## 4. Discussion

In the initial study and then a second larger, confirmatory single site, cross-over designed study, both gummy and tablet preparations had a peak vitamin D_3_ concentration around 10 h. In the confirmatory study, there were statistically significant differences noted in bioavailability, with greater bioavailability with the gummy preparation compared to the tablet preparation. The data suggest that the gummy vitamin D_3_ preparation has greater bioavailability than the tablet preparation and leads to better gastrointestinal absorption as evidenced by higher total circulating vitamin D_3_ concentrations at serial time points following ingestion.

The formulation of a supplement includes the active ingredient—in this case vitamin D_3_—and excipients, such as vehicle(s) and fillers that help stabilize the active ingredient(s) and aid in the administration and absorption of the active ingredient(s). The bioavailability of a lipophilic agent such as vitamin D is dependent on the proportion of the lipophilic agent released from its food matrix into the salivary and gastric juices, its release and solubilization into micelles that must be accessible for absorption by enterocytes, and its release into the circulation without being metabolized [12]. Coating on tablet or gummies can affect the dissolution of the food and thus absorption. Other factors commonly listed that affect bioavailability include competing foods and bile acid secretion [12].

In the present study, the vehicle for the gummies was syrup/sucrose/gelatin/pectin compared to the commercial tablet label, which lists cellulose gel/maltodextrin/gelatin/cornstarch in the tablets. Given its shorter half-life, using the parent compound vitamin D for the indicator of bioavailability for the two vitamin preparations for each participant allowed a more precise measurement of potential intestinal absorption differences. Reliance of earlier studies on 25(OH)D concentration with its longer t½ life of 2–3 weeks does not capture the rapid differences in absorption between various vitamin D preparations that is captured by measuring vitamin D itself, with its t½ of 12–24 h [13].

Absorption of vitamin D from the gastrointestinal tract is thought to be mainly passive [14,15,16], but its absorption can be influenced by other foods and substances such as cholesterol and tocopherol (vitamin E) and to be protein-mediated at lower concentrations [12]. Reboul et al., in their study to establish the details of vitamin D transport across the enterocyte, used a well-established Caco-2 TC-7 cell model that yields results consistent with human in vivo data (17). The investigators studied cholecalciferol in a range that corresponded to the concentration in mixed micelles recovered from the duodenum after digestion (0.1–0.5 µM) to mimic a typical dietary situation and up to 10 µM to mimic pharmacological conditions. The lower range of saturable uptake suggested protein mediated uptake, but at pharmacological concentrations, cholecalciferol uptake was linear, consistent with passive diffusion [17], confirming earlier studies by Hollander et al. [15,16]. Competition studies with cholesterol and tocopherol (vitamin E) decreased cholecalciferol uptake. Further cholecalciferol uptake was inhibited by a specific inhibitor of cholesterol transport, SR-B1, which is involved in both lipid uptake and efflux. These findings, confirmed by mouse intestinal explant studies [17], have implications for understanding potential differences between supplement preparations.

The bioavailability of supplements is partially dependent on the dissolution of the supplement preparation, which determines absorption. Coating of the supplement also affects dissolution. In the present two studies, the participants did not have competing foods at the time of the administration and were fed a standardized breakfast after administration of the vitamin D formulations. As a potential explanation for the differences in bioavailability between gummy and tablet formulations, however, one also must consider the digestive processes that begin in the mouth. Gummies are chewed and would begin their dissolution in the mouth when combined with saliva. This process of dissolution would continue in the stomach, with further dissolution in the small intestine, particularly in the second part of the duodenum, where the pancreas deposits bile acids for fat digestion [12]. The dissolution differences would affect C_max_, thereby leading to greater bioavailability of gummies compared to tablets. Some of the dissolving gummies theoretically could be absorbed sublingually as well [18]: Not often considered as a mechanism of absorption, with the potential to increase bioavailability, sublingual digestion and absorption may be a possible mechanism of increased absorption of gummies compared to tablets, which are swallowed whole [18]. Sublingual absorption is influenced by lipophilicity of the drug or supplement, with greater absorption with higher lipid solubility than that required for GI absorption. Any agent absorbed in this manner bypasses the liver and also is protected from degradation due to the pH and digestive enzymes of the middle GI tract [18]. Further study would be necessary to examine this potential site of vitamin D absorption with respect to the gummy preparation.

Other studies point to the importance of the vehicles in which vitamin D is matrixed. In a meta-analysis by Grossmann and Tangpricha, comparing the bioequivalence of vitamin D in vehicles of powders, lipids/oil-based or ethanol preparations, the authors found that the oil-soluble vehicles produced the greatest rate of change in mean 25(OH)D per 100,000 IU of vitamin D, followed by powder-based vehicles and vitamin D dissolved in ethanol, but there was considerable variability in outcomes reported [19]. A study by Khazai et al. involving cystic fibrosis patients found better absorption in that population with a powder vehicle (D_3_) than an oil-soluble vehicle (D_2_), likely reflecting issues with lipid absorption in that population [20], but the forms of vitamin D were not the same. In another study by the same group, also involving cystic fibrosis patients, comparing D_3_ bioavailability of the powder vs. oil-soluble vehicle, the authors again found the powder to have greater bioavailability than the oil preparation, validating their earlier findings using cholecalciferol (vitamin D_3_) in both preparations [21].

The strengths of the two studies presented here were that each participant served as her/his own control in a cross-over design and that the analyzing laboratories were blinded to intervention. The first study served to provide the required data to determine the necessary sample size to achieve adequate power to show differences in bioavailability. In addition, the participants were of diverse racial and ethnic backgrounds, allowing more generalizability of the data. Unlike the majority of published bioequivalence studies, this study reported changes in the parent compound vitamin D, with its shorter half-life than 25(OH)D, which allowed a more precise measurement of potential intestinal absorption differences. The limitations of the two studies were that the investigation team was not blinded to the intervention when the participants were dosed; however, those drawing the participant blood samples and the laboratories were blinded to intervention as was the laboratory measuring blood vitamin D_3_ concentrations.

In summary, in both the initial pilot study and the subsequent larger, single-site cross-over design study, a gummy preparation appeared to have greater bioavailability compared to the tablet preparation. These findings have implications for achieving targeted circulating vitamin D levels, which have been linked with certain health outcomes.

## Figures and Tables

**Table 1:** Inclusion and exclusion criteria.

**Inclusion Criteria**
Ages 18–45 years Sexes: males and females Females not of childbearing potential (i.e., hysterectomy, oophorectomy, bilateral tubal ligation, or postmenopausal) or females of childbearing potential that agree to use a medically approved method of birth control such as hormonal contraceptives, double-barrier, non-hormonal intrauterine devices, or non-heterosexual lifestyle or agree to use contraception if planning on changing to heterosexual partner(s) and/or vasectomy of partner BMI 18.5 to 29.9 kg/m^2^ Agree to maintain current level of physical activity throughout the study Agree to wear sunblock of at least SPF 45 during the study and not to have excessive sun exposure (no more than 1 h without sunblock)
**Exclusion Criteria**
Women who are pregnant, to be determined by UPT (urine pregnancy test), breastfeeding, or planning to become pregnant during the course of the study Duodenal ulcer or gastric ulcer, gastritis, hiatus hernia, or GERD within the past 3 months Significant gastrointestinal disease, history of malabsorption, or history of irritable bowel syndrome and related disorders Unstable medical conditions as determined by the principal investigator Clinically significant abnormal laboratory results on CBC or BMP at screening Cancer chemotherapy/radiation treatment within the 3 months prior to enrollment Metabolic disease History of kidney stones Use of prescription or over the counter products known to interact with vitamin D within 72 h of randomization and during the trial such as aspirin and NSAIDs, aluminum, iron, and proton pump inhibitors Use of acute over the counter medication within 72 h of test product dosing Smokers Consumption of more than 2 alcoholic drinks per day Drug abuse within the past year Use of medicinal marijuana Immunocompromised individuals such as individuals that have undergone organ transplantation or individuals diagnosed with human immunodeficiency virus (HIV) Individuals who have planned surgery during the course of the trial Use of St. John’s wort in the last 30 days before randomization and during the study Use of vitamin D, multivitamins containing vitamin D, foods or beverages fortified with vitamin D and other natural healthy products containing vitamin D, or consumption of grapefruit/grapefruit juice within 14 days of randomization and during the study Use of anticoagulants, barbiturates, tetracycline antibiotics, beta-blockers, cyclosporine, prednisone, tricyclic antidepressants, diuretics, and nitrate medications History of blood/bleeding disorders Anemia of any etiology defined as hemoglobin < 14.0 mg/dL for males and < 12.3 mg/dL for females Blood donation in the past 3 months, or individuals planning to donate blood during the study or within 30 days of completion of study Participation in a clinical research trial within 30 days prior to randomization Allergy or sensitivity to any ingredient in supplements provided during the study Individuals who are cognitively impaired and/or who are unable to give informed consent

**Table 2:** Participant sociodemographic and clinical characteristics in Study 1 (*n* = 9).

Characteristic	
Gender	5 females (5 Caucasian/white)
	4 males (3 Caucasian/white, 1 Hispanic)
Race/Ethnicity	8 Caucasian/white
	1 Hispanic
Mean Age of Subjects, Years ± SD (Range, years)	27.1 ± 3.7
	(22–34)
Mean Hemoglobin ± SD at Screening (g/dL)	Overall: 13.8 ± 1.2
	Females: 12.8 ± 0.4
	Males: 15.0 ± 0.6
Total Circulating 25(OH)D ± SD at Screening (ng/mL)	Overall: 30.1 ± 5.2
	Females: 31.1 ± 5.5
	Males: 28.8 ± 5.2

**Table 3:** Study 1 (*n* = 9). Mean ± SD changes from baseline vitamin D_3_ blood concentrations (ng/mL) at each time point measured.

D_3_			
Hour	Gummy Mean ± SD	Tablet Mean ± SD	Adjusted Effect Size *
3	8.0 ± 6.7	7.8 ± 4.7	0.06
6	25.0 ± 14.4	18.2 ± 8.3	0.54
10	33.8 ± 11.2	27.0 ± 7.0	0.87
24	24.1 ± 6.3	19.9 ± 6.0	1.08
48	12.7 ± 3.0	12.0 ± 3.2	0.34

* Effect sizes were adjusted for baseline concentration.

**Table 4:** Participant sociodemographic and clinical characteristics in study 2 (*n* = 31).

Characteristic	
Gender	17 females (13 Caucasian/white and 4 Hispanic)
	14 males (9 Caucasian/white, 2 Hispanic, and 3 Asian/Middle Eastern)
Race/Ethnicity	22 Caucasian/white
	6 Hispanic
	3 Asian/Middle Eastern
Mean Age of Subjects, Years ± SD (Range, years)	28.3 ± 5.6
	(22–43)
Mean Hemoglobin ± SD at Screening (g/dL)	Overall: 14.1 ± 1.4
	Females: 13.2 ± 1.0
	Males: 15.2 ± 0.9
Total Circulating 25(OH)D ± SD at Screening (ng/mL)	Overall: 31.9 ± 7.3
	Females: 33.3 ± 8.2
	Males: 32.3 ± 7.4

**Table 5:** Study 2 (*n* = 31). Mean ± SD changes from baseline vitamin D_3_ blood concentrations (ng/mL) at each time point measured.

D_3_			
Hour	Gummy Mean ± SD	Tablet Mean ± SD	Adjusted Effect Size *
3	16.4 ± 12.6	6.1 ± 5.3	1.02
6	36.7 ± 12.8	16.3 ± 5.6	1.75
10	43.5 ± 11.2	19.8 ± 4.2	2.14
24	29.9 ± 6.4	14.4 ± 2.9	2.75
48	15.4 ± 3.6	7.8 ± 2.1	2.30

* Effect sizes were adjusted for baseline concentration.

## Abbreviations

25-hydroxy-vitamin D	25(OH)D
Area under the curve	AUC
Body mass index	BMI
Cholecalciferol:vitamin D_3_	vitD
Concentration max	C_max_
Hour	h
International Unit	IU
Medical University of South Carolina	MUSC
Minutes	min
Time peak concentration	T_max_
Revolutions per minute	rpm
